# Investigating Doxorubicin’s mechanism of action in cervical cancer: a convergence of transcriptomic and metabolomic perspectives

**DOI:** 10.3389/fgene.2023.1234263

**Published:** 2023-08-28

**Authors:** Zhuo Huang, Huining Jing, Juanjuan Lv, Yan Chen, YuanQiong Huang, Shuwen Sun

**Affiliations:** ^1^ Department of Pediatrics, West China Second University Hospital, Sichuan University, Chengdu, China; ^2^ Key Laboratory of Birth Defects and Related Diseases of Women and Children, Ministry of Education, West China Second University Hospital, Sichuan University, Chengdu, China; ^3^ Department of Medical Genetics, West China Second University Hospital, Sichuan University, Chengdu, China; ^4^ Department of Obstetrics and Gynecology, West China Second University Hospital, Sichuan University, Chengdu, China; ^5^ Department of Oncology, Luzhou Hospital of Traditional Chinese Medicine, Luzhou, China

**Keywords:** cervical cancer, Doxorubicin, transcriptomics, metabolomics, ANKRD18B, L-Ornithine, multi-omics integration

## Abstract

**Introduction:** Cervical cancer remains a significant global health burden, and Doxorubicin is a crucial therapeutic agent against this disease. However, the precise molecular mechanisms responsible for its therapeutic effects are not fully understood.

**Methods:** In this study, we employed a multi-omics approach that combined transcriptomic and metabolomic analyses with cellular and *in vivo* experiments. The goal was to comprehensively investigate the molecular landscape associated with Doxorubicin treatment in cervical cancer.

**Results:** Our unbiased differential gene expression analysis revealed distinct alterations in gene expression patterns following Doxorubicin treatment. Notably, the ANKRD18B gene exhibited a prominent role in the response to Doxorubicin. Simultaneously, our metabolomic analysis demonstrated significant perturbations in metabolite profiles, with a particular focus on L-Ornithine. The correlation between ANKRD18B gene expression and L-Ornithine levels indicated a tightly controlled gene-metabolite network. These results were further confirmed through rigorous cellular and *in vivo* experiments, which showed reductions in subcutaneous tumor size and significant changes in ANKRD18B, L-Ornithine, and Doxorubicin concentration.

**Discussion:** The findings of this study underscore the intricate interplay between transcriptomic and metabolomic changes in response to Doxorubicin treatment. These insights could have implications for the development of more effective therapeutic strategies for cervical cancer. The identification of ANKRD18B and L-Ornithine as key components in this process lays the groundwork for future research aiming to unravel the complex molecular networks that underlie Doxorubicin’s therapeutic mechanism. While this study provides a solid foundation, it also highlights the necessity for further investigation to fully grasp these interactions and their potential implications for cervical cancer treatment.

## Introduction

Cervical cancer, an intricate health concern worldwide, predominantly afflicts women and stands as the fourth most common malignancy in the female population (Ginsburg et al., 2017). The global burden of cervical cancer is colossal, with an estimated 604,000 new cases and around 342,000 deaths reported in 2022, according to the World Health Organization (Tan et al., 2022). Despite the advent of novel diagnostic techniques and preventative strategies such as the human papillomavirus (HPV) vaccination, the morbidity and mortality rates remain daunting, particularly in low- and middle-income countries where healthcare resources and infrastructure are limited (Bosch et al., 2013). It is widely acknowledged that persistent infection with certain types of human papillomavirus (HPV) is the most important risk factor for cervical cancer. Indeed, HPV is detected in more than 90% of cervical cancer cases, with HPV types 16 and 18 being the most prevalent. HPV, a small DNA virus, has over 100 types, of which about 40 can infect the genital tract. Among these, approximately 15 types are considered high-risk for the development of cervical cancer and other anogenital cancers (Ahmed et al., 2023). Prolonged infection with these high-risk types, particularly HPV 16 and HPV 18, can lead to the formation of precancerous lesions, which may progress to invasive cervical cancer if not treated (Cascardi et al., 2022). Despite the availability of prophylactic HPV vaccines, cervical cancer incidence remains high due to factors such as limited vaccine coverage and the long period between HPV infection and cancer development. Furthermore, the vaccines do not have therapeutic effects on existing HPV infections or HPV-associated lesions. As such, understanding the molecular mechanisms underlying cervical cancer progression and response to treatment, as explored in this study, is of utmost importance.

The standard therapeutic approach for cervical cancer encompasses a combination of surgery, radiation therapy, and chemotherapy, tailored according to the stage and extent of the disease (Yessaian et al., 2004). Doxorubicin, a potent anthracycline chemotherapy drug, has been employed as a primary or adjuvant treatment modality in various stages of cervical cancer (Koning et al., 2010). It operates primarily by intercalating into DNA, inhibiting the topoisomerase II enzyme, thus impeding DNA replication and transcription, culminating in cell death. Nevertheless, the precise molecular and metabolic pathways influenced by Doxorubicin in cervical cancer remain to be comprehensively elucidated. Recent advancements in high-throughput technologies have made it feasible to explore the complex landscape of biological systems at multiple levels, from genes to metabolites (Yessaian et al., 2004).

Transcriptomics and metabolomics, two integral components of systems biology, provide profound insights into the functional elements of the genome and the downstream metabolic processes, respectively (Zampieri et al., 2019; Jamil et al., 2020). These methodologies have empowered the scientific community to probe into the molecular intricacies of disease mechanisms, drug responses, and personalized therapeutics (Naylor and Chen, 2010; Beckmann and Lew, 2016). In the realm of cervical cancer, an integrative analysis of transcriptomic and metabolomic data may unveil the precise molecular and metabolic alterations induced by Doxorubicin. Such comprehensive understanding could aid in the optimization of treatment strategies, identification of potential therapeutic targets, and prediction of treatment response (Fleisher et al., 2017; Niu et al., 2019). This study leverages the synergistic potential of transcriptomics and metabolomics to investigate the mechanism of action of Doxorubicin in cervical cancer, paving the way for innovative and personalized treatment options in the future.

## Materials and methods

### Public data retrieval and preprocessing

Transcriptomic data for this study were obtained from the publicly available dataset GSE160234 (Demir et al., 2021). The experimental design encompassed HeLa cells that were incubated for 6 days without treatment (group Normal, *n* = 3), treated with 300 nM doxorubicin for 72 h and then incubated for an additional 72 h without treatment (group Cancer, *n* = 3), or treated with 300 nM doxorubicin for 72 h. These data were preprocessed and normalized according to the standard pipelines to ensure the accuracy and reliability of subsequent analyses.

### Metabolomics analysis

For the metabolomic study, cells under the same treatment conditions were harvested, and metabolites were extracted using a cold methanol-acetonitrile-water solution (2:2:1 v/v/v). The extracts were then analyzed using an Agilent 1290 Infinity II LC system coupled with an Agilent 6545 Q-TOF mass spectrometer (Agilent Technologies, Santa Clara, CA, United States). The chromatographic separation was carried out on a Waters ACQUITY UPLC BEH Amide column (2.1 mm × 100 mm, 1.7 μm). The raw data were processed and analyzed using Agilent MassHunter Qualitative Analysis software (B.06.00).

### Quantitative polymerase chain reaction (qPCR)

Quantitative polymerase chain reaction (qPCR) was performed to validate the expression of the ANKRD18B gene. RNA was extracted from the cells using the RNeasy Mini Kit (Qiagen, Valencia, CA, United States), and cDNA was synthesized using the iScript cDNA Synthesis Kit (Bio-Rad, Hercules, CA, United States). Real-time PCR was performed using the SYBR Green Master Mix (Applied Biosystems, Foster City, CA, United States) on a StepOnePlus Real-Time PCR System (Applied Biosystems, Foster City, CA, United States). The primer sequences for ANKRD18B were: forward, 5′- CTC​GCT​CTA​TCA​CCA​GTC​TGG​A -3′; reverse, 5′- ATG​GTC​GCA​TGT​GCC​TGT​TGT​C -3′. Beta-actin was used as the reference gene, with the following primer sequences: forward, 5′- CAC​CAT​TGG​CAA​TGA​GCG​GTT​C -3′; reverse, 5′- AGG​TCT​TTG​CGG​ATG​TCC​ACG​T -3′.

### *In vivo* mouse model experiments

The nude mice were inoculated subcutaneously with HeLa cells (ATCC^®^ CCL-2™) to generate xenograft tumors. Post-inoculation, the mice were intraperitoneally treated with either doxorubicin (CAS 25316-40-9, Sigma-Aldrich, St. Louis, MO, United States) at varying doses between 2 and 3 mg/kg, or normal saline (CAS 7647-14-5, Sigma-Aldrich, St. Louis, MO, United States) at doses between 10 and 12 mg/kg, twice a week. The treatments started from the seventh day post-inoculation, and the experiment lasted for 28 days.

### Statistical analysis

All statistical analyses were performed using the R programming language (version 4.0.2). Descriptive statistics were used to summarize the transcriptomics and metabolomics data. The Normal and Cancer groups were compared using Student’s *t*-test for normally distributed data or the Mann-Whitney U test for non-normally distributed data. A *p*-value of less than 0.05 was considered statistically significant. Correlation analyses between the transcriptomics and metabolomics data were conducted using Pearson’s correlation coefficient or Spearman’s rank correlation coefficient as appropriate. The most significant genes and metabolites were selected based on the correlation coefficients. Differential expression analyses for the transcriptomics data were conducted using the DESeq2 package (Love et al., 2014). Genes with an adjusted *p*-value (Benjamini-Hochberg procedure) less than 0.05 and a log2 fold-change greater than 1 or less than −1 were considered differentially expressed. For the qPCR and *in vivo* mouse model experiment data, one-way analysis of variance (ANOVA) followed by Tukey’s multiple comparisons test was used to compare the groups. Data visualization was performed using the ggplot2 package in R. Heatmaps were generated using the pheatmap package (Kolde and Kolde, 2018). Boxplots were used to visualize the expression levels of the most significant genes and metabolites.

## Results

### Revealing altered gene expression landscape in cervical cancer cells treated with Doxorubicin: a comprehensive transcriptomic analysis

**FIGURE 1:**
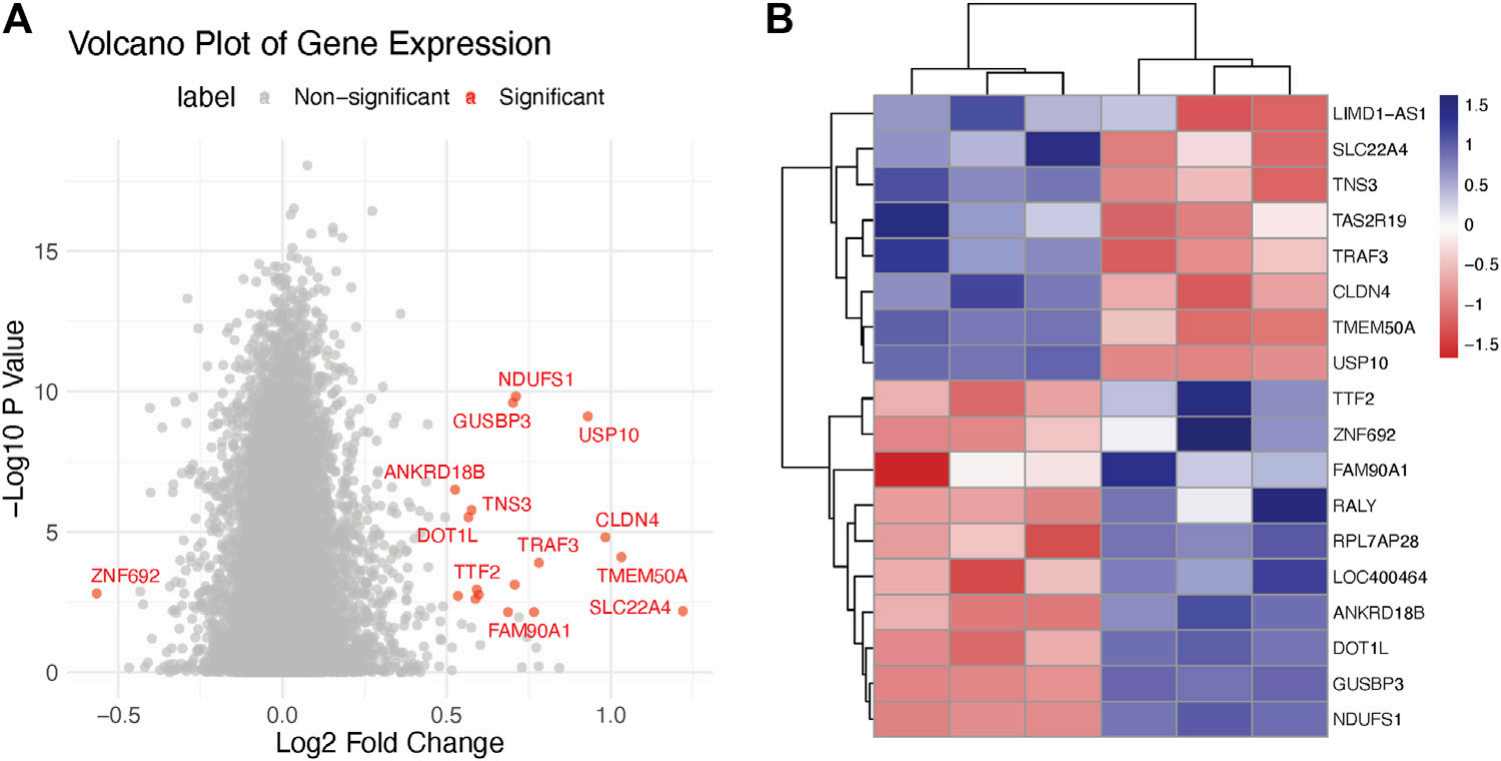
Transcriptomics analysis of doxorubicin-treated and control Hela cells. **(A)** Volcano plot illustrating the differential expression of genes in Hela cells treated with doxorubicin versus control. The X-axis represents the log2 fold change (FC) and the Y-axis represents the −log10 adjusted *p*-values. **(B)** Heatmap showing the clustering of significantly differentially expressed genes (DEGs) in doxorubicin-treated and control Hela cells.

As part of our endeavor to characterize the molecular mechanisms that underpin the therapeutic effect of Doxorubicin on cervical cancer, we began with a deep dive into the transcriptomic changes it instigates. We carried out an unbiased differential gene expression analysis to discern the molecular patterns associated with Doxorubicin treatment. Our analysis led us to the discovery of several genes that displayed marked expression differences between the Normal and Cancer groups. The ensuing volcano plot—Figure 1A—brings into stark relief the multitude of differentially expressed genes. Notably, the distribution of these genes followed an interesting pattern, suggesting potential stratification of gene expression alterations. Moreover, to zero in on the most significantly altered genes, we constructed a heatmap using those with an adjusted *p*-value < 0.05 and |log2 fold-change| > 1 (Figure 1B). This analysis unveiled clusters of genes with distinct expression patterns, giving us valuable insights into the potential molecular programs disrupted by Doxorubicin (Supplementary Table S1).

### Doxorubicin’s therapeutic action is accompanied by perturbations in metabolite profiles: an in-depth metabolomic analysis

**FIGURE 2:**
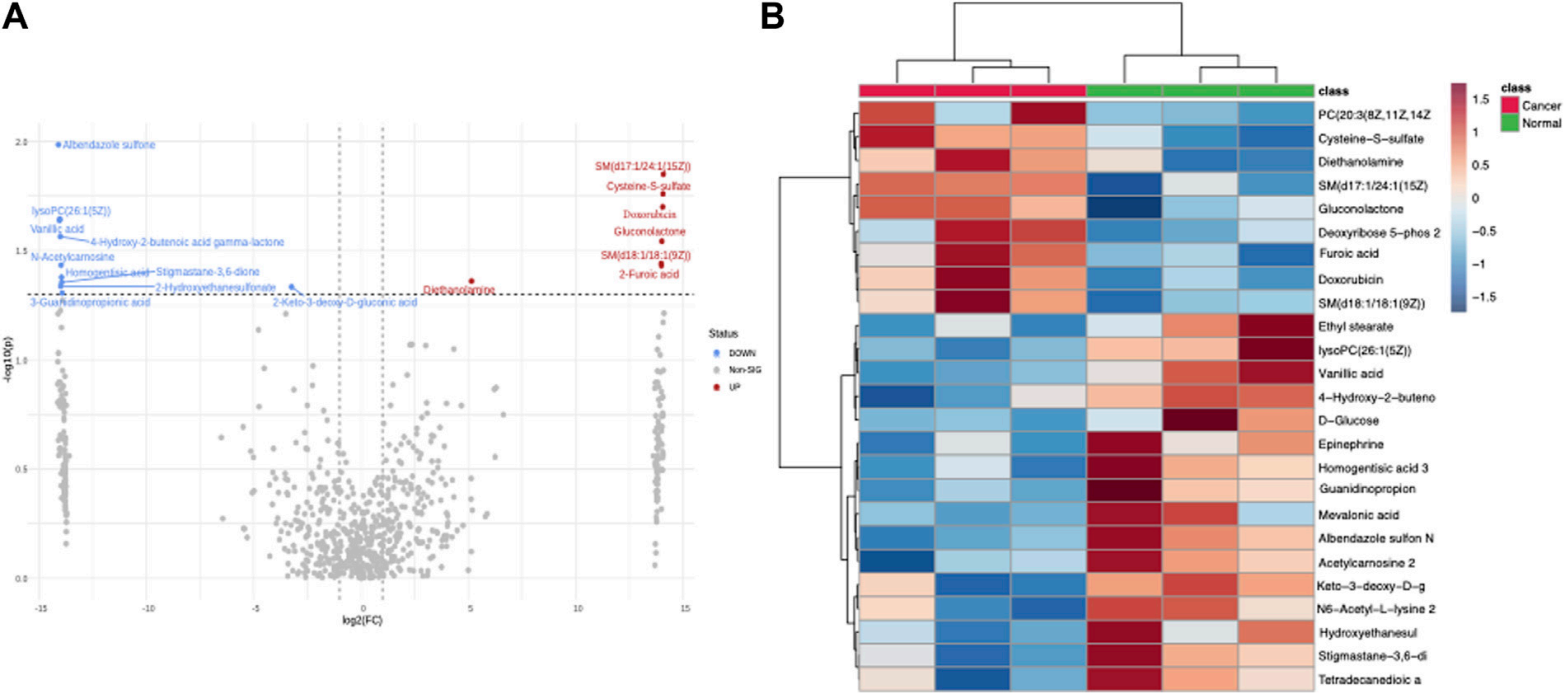
Metabolomics analysis of doxorubicin-treated and control Hela cells. **(A)** Volcano plot depicting the differential metabolite levels in Hela cells treated with doxorubicin versus control. The X-axis shows the log2 FC and the Y-axis represents the −log10 adjusted *p*-values. **(B)** Heatmap demonstrating the clustering of significantly different metabolites in doxorubicin-treated and control Hela cells.

Concurrent with our transcriptomic analysis, we undertook an exhaustive metabolomic analysis to unravel any accompanying metabolic perturbations associated with Doxorubicin treatment. Interestingly, like our transcriptomic analysis, many metabolites exhibited significantly differential levels between the Normal and Cancer groups. The ensuing volcano plot (Figure 2A) clearly illustrates this widespread metabolic perturbation. To provide a finer granularity of these metabolic changes, we charted a heatmap using the most significantly altered metabolites. The heatmap in Figure 2B depicts an intricate metabolic landscape, suggesting a complex remodeling of metabolic pathways in response to Doxorubicin treatment (Supplementary Table S2).

### A symbiotic relationship between transcriptomic and metabolomic alterations: integrated correlation analysis

**FIGURE 3:**
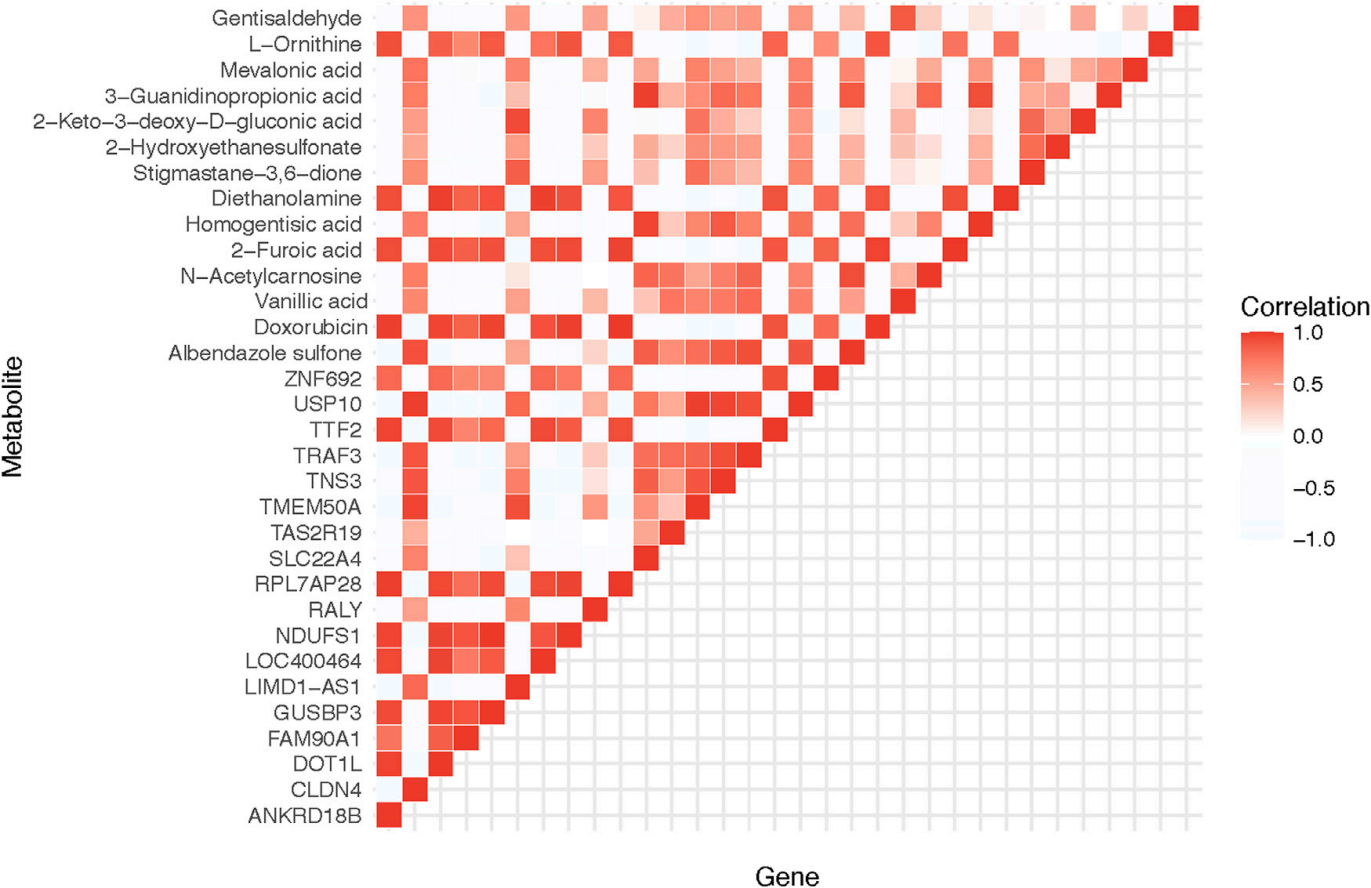
Correlation analysis of significantly differential genes and metabolites. The heatmap demonstrates the correlation between the DEGs and the significantly different metabolites. ANKRD18B and L-Ornithine are found to have a significant correlation.

With the wealth of data generated from our omics analyses, we sought to tease apart any possible relationships between the transcriptomic and metabolomic changes. In doing so, we uncovered a strong correlation between the gene ANKRD18B and the metabolite L-Ornithine, both of which showed marked changes in response to Doxorubicin treatment. As shown in Figure 3, this result illustrates the intimate interplay between gene expression and metabolite levels, potentially pointing towards a tightly controlled gene-metabolite network affected by Doxorubicin.

### From cells to mice: experimental validation of omics findings

**FIGURE 4:**
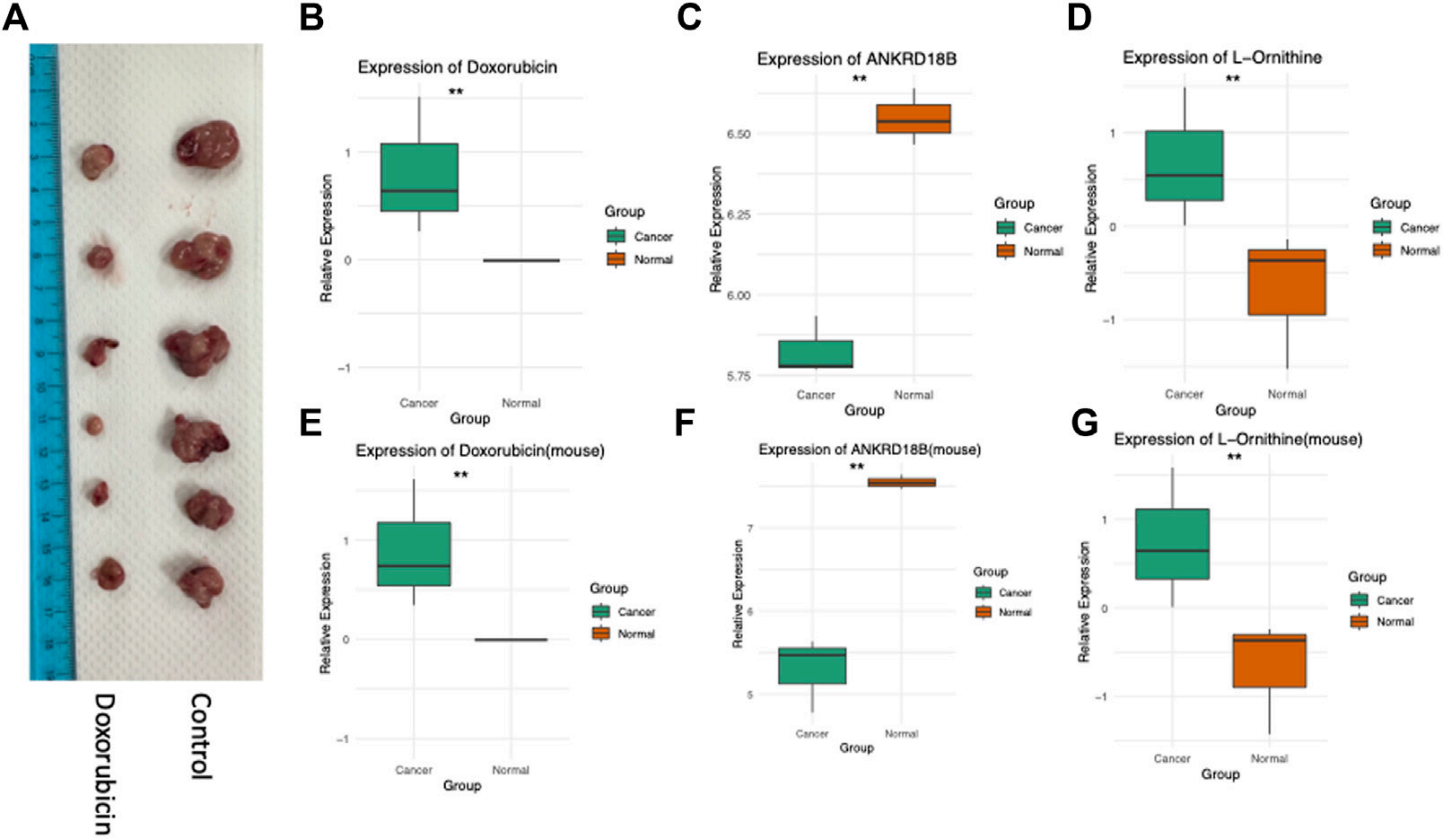
Experimental validation in cells and *in vivo*. **(A)** Doxorubicin inhibits the growth of Hela cell-derived subcutaneous tumors in mice. **(B)** ANKRD18B gene expression, **(C)** doxorubicin concentration, and **(D)** L-Ornithine concentration in cell cultures. **(E)** ANKRD18B gene expression, **(F)** doxorubicin concentration, and **(G)** L-Ornithine concentration in tumor tissues. *p < 0.05; **p < 0.01.

Complementing our multi-omics analyses, we conducted a series of rigorous cellular and *in vivo* experiments to validate our findings. The effect of Doxorubicin treatment on tumor growth was assessed in a mouse model, where we observed a significant decrease in subcutaneous tumor size (Figure 4A). Furthermore, cellular and tissue experiments corroborated the crucial roles of ANKRD18B and L-Ornithine in mediating the action of Doxorubicin. As evident from Figures 4B, E, ANKRD18B expression levels were significantly different in both the cell and tissue experiments, aligning with our omics data. Similarly, the intracellular concentrations of Doxorubicin (Figures 4C, F) and L-Ornithine (Figures 4D, G) were also found to vary significantly in line with the corresponding omics results. Collectively, these data provide compelling evidence for the involvement of ANKRD18B and L-Ornithine in the therapeutic mechanism of Doxorubicin, thus enriching our understanding. In our analysis of the TCGA-CESC (Cervical Squamous Cell Carcinoma and Endocervical Adenocarcinoma) dataset, ANKRD18B emerged as a gene of significant interest (Supplementary Figure S1). We found that the expression of ANKRD18B was significantly elevated in tumor tissues compared to their normal counterparts (*p* < 0.05). This upregulation in tumor tissues suggests a potential role of ANKRD18B in cervical cancer progression. However, when we examined the impact of ANKRD18B expression on overall survival, the results were not statistically significant (logrank *p* = 0.45).

## Discussion

In this study, we embarked on a comprehensive investigation to elucidate the molecular underpinnings of the therapeutic action of Doxorubicin on cervical cancer. Our approach involved an integration of transcriptomic and metabolomic analyses, paired with rigorous cellular and *in vivo* experiments. This multi-pronged strategy led us to the discovery of ANKRD18B and L-Ornithine as significant players in the Doxorubicin treatment response. The observed positive correlation between ANKRD18B and L-Ornithine is of special interest and warrants further investigation to unravel the intricacies of their joint impact on the disease process.

ANKRD18B, part of the Ankyrin repeat domain-containing protein family, has been previously implicated in various biological processes, albeit the exact mechanism of action of ANKRD18B is not fully understood (Sundar et al., 2017). Recent studies have shed light on the potential role of ANKRD18B in cellular processes such as cell cycle regulation and apoptosis, both of which are key mechanisms exploited by chemotherapeutic drugs like Doxorubicin (Al-Alem, 2011). The marked upregulation of ANKRD18B in response to Doxorubicin treatment, as revealed by our transcriptomic analysis, points towards a potential mechanistic role of this gene in the anti-tumor action of Doxorubicin. Moreover, the fact that its expression pattern is in sync with Doxorubicin concentration underscores its potential importance in Doxorubicin-mediated therapeutic effect. Although the exact role of ANKRD18B in this process is still unclear, its significant alteration following Doxorubicin treatment, as shown in our study, suggests that it may be implicated in the response to therapy in HPV-positive cervical cancers. On the other hand, L-Ornithine, a key player in the urea cycle, has been linked to multiple cancer-related processes (You et al., 2018). Several studies have implicated aberrant metabolism, including alterations in the urea cycle, as a hallmark of cancer (Erbaş et al., 2015; Wang et al., 2022). In cervical cancer, metabolic reprogramming often manifests as alterations in amino acid metabolism, of which the urea cycle is an integral part. Thus, our finding of significant alterations in L-Ornithine levels in response to Doxorubicin treatment is noteworthy. Given the critical role of metabolic remodeling in cancer progression and response to therapy, the observed L-Ornithine changes might signify an important metabolic response to Doxorubicin. Additionally, the fact that L-Ornithine levels mirrored those of ANKRD18B and Doxorubicin concentration points to a potential gene-metabolite network at play.

Interestingly, the positive correlation between ANKRD18B and L-Ornithine highlights a potential intricate interplay between gene expression and metabolite levels. Such an interaction could potentially contribute to the complex adaptive response of cancer cells to chemotherapy, thereby impacting the therapeutic outcome. The discovery of this correlation underscores the importance of integrative multi-omics analyses in unveiling complex molecular networks. While it necessitates further exploration, it provides a promising direction for future research aimed at a more comprehensive understanding of the therapeutic mechanism of Doxorubicin.

Our study is still with some limitations. While we have uncovered promising candidates that might be pivotal in the therapeutic response to Doxorubicin, the precise nature of their interaction and the resulting molecular cascade remains to be elucidated. Future work should focus on deciphering these molecular networks using techniques like gene knockdown or overexpression studies and metabolic flux analysis. Nonetheless, our findings lay a solid foundation for future research in this direction and can potentially aid the design of improved therapeutic strategies. Although our multi-omics approach has provided valuable insights into the molecular mechanisms of Doxorubicin, it is important to note some limitations of our study. One of these is the inability of our metabolomics equipment to differentiate between the D and L forms of metabolites. This is a significant limitation as the D and L forms can have different biological activities. Future studies with equipment capable of distinguishing between these forms would provide a more comprehensive understanding of the changes in the metabolome following Doxorubicin treatment.

In conclusion, our study highlights the intricate changes in gene expression and metabolic profiles in response to Doxorubicin treatment in cervical cancer. We provide compelling evidence pointing towards a crucial role of ANKRD18B and L-Ornithine in mediating the action of Doxorubicin. While our findings paint a complex picture of the molecular landscape associated with Doxorubicin treatment, they open new avenues for a deeper.

## Data Availability

The raw data supporting the conclusion of this article will be made available by the authors, without undue reservation.

## Supplementary material

SUPPLEMENTARY FIGURE S1
**(A)** Differential expression of ANKRD18B in TCGA-CESC dataset. This bar graph represents the expression levels of ANKRD18B in paired tumor (T) and normal (N) tissues from the TCGA-CESC dataset. The expression of ANKRD18B was significantly elevated in tumor tissues compared to their normal counterparts (*p* < 0.05). Error bars denote standard error of the mean (SEM). **(B)** Kaplan-Meier survival analysis of cervical cancer patients based on ANKRD18B expression. The survival curves represent the overall survival of cervical cancer patients stratified by the expression level of ANKRD18B (high vs. low). Despite the observed difference in survival times, the log-rank test did not reveal a significant difference (*p* = 0.45).
