# Supplementary figures and images for: Investigating Doxorubicin’s mechanism of action in cervical cancer: a convergence of transcriptomic and metabolomic perspectives
Source: Front Genet. 2023 Aug 28;14:1234263. doi: 10.3389/fgene.2023.1234263 (PMC10494242; doi:10.3389/fgene.2023.1234263)

A

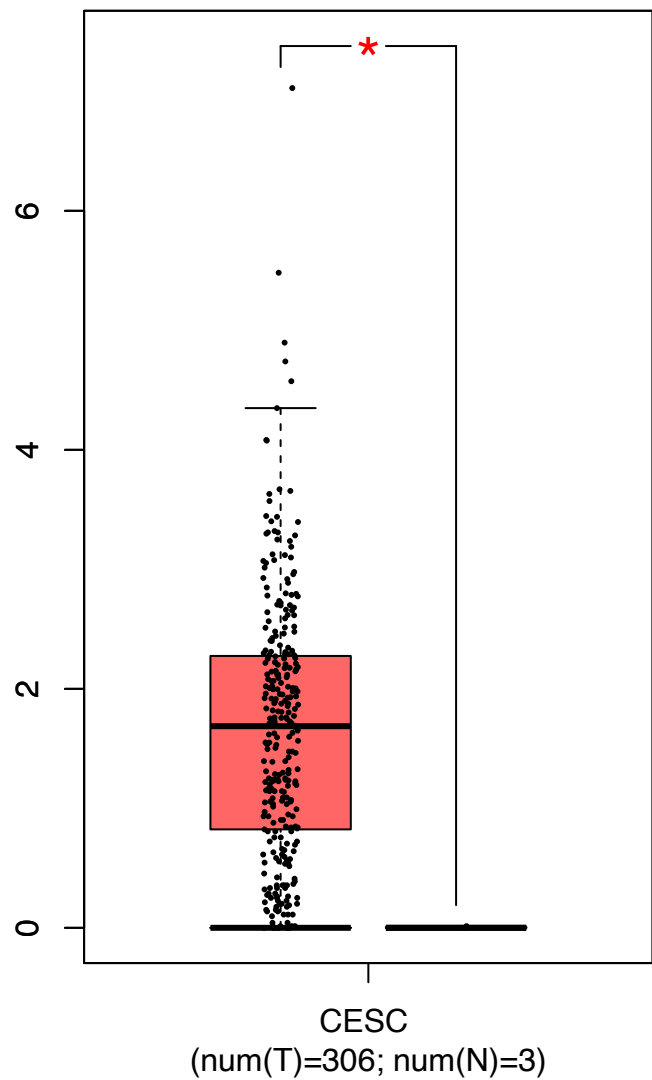

B

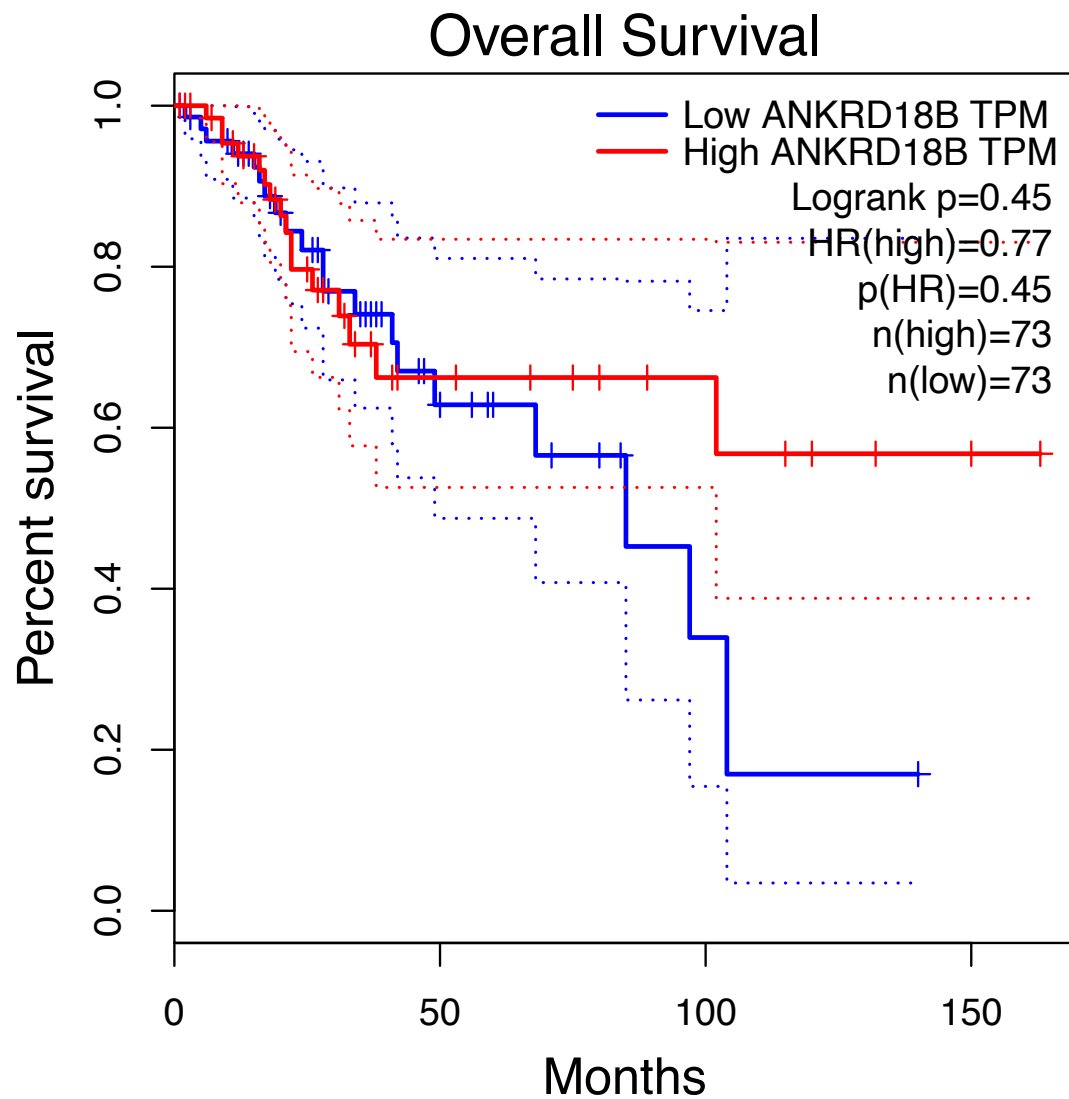

Supplement: Supplementary file 3 [file Image1.pdf]
